# Exploring the Ecological Validity of Thinking on Demand: Neural Correlates of Elicited vs. Spontaneously Occurring Inner Speech

**DOI:** 10.1371/journal.pone.0147932

**Published:** 2016-02-04

**Authors:** Russell T. Hurlburt, Ben Alderson-Day, Simone Kühn, Charles Fernyhough

**Affiliations:** 1 Psychology, University of Nevada Las Vegas, Las Vegas, Nevada, United States of America; 2 Psychology, Durham University, Durham, United Kingdom; 3 Center for Lifespan Psychology, Max Planck Institute for Human Development, Berlin, Germany; Birkbeck College, UNITED KINGDOM

## Abstract

Psychology and cognitive neuroscience often use standardized tasks to elicit particular experiences. We explore whether elicited experiences are similar to spontaneous experiences. In an MRI scanner, five participants performed tasks designed to elicit inner speech (covertly repeating experimenter-supplied words), inner seeing, inner hearing, feeling, and sensing. Then, in their natural environments, participants were trained in four days of random-beep-triggered Descriptive Experience Sampling (DES). They subsequently returned to the scanner for nine 25-min resting-state sessions; during each they received four DES beeps and described those moments (9 × 4 = 36 moments per participant) of spontaneously occurring experience. Enough of those moments included spontaneous inner speech to allow us to compare brain activation during spontaneous inner speech with what we had found in task-elicited inner speech. ROI analysis was used to compare activation in two relevant areas (Heschl’s gyrus and left inferior frontal gyrus). Task-elicited inner speech was associated with decreased activation in Heschl’s gyrus and increased activation in left inferior frontal gyrus. However, spontaneous inner speech had the opposite effect in Heschl’s gyrus and no significant effect in left inferior frontal gyrus. This study demonstrates how spontaneous phenomena can be investigated in MRI and calls into question the assumption that task-created phenomena are often neurophysiologically and psychologically similar to spontaneously occurring phenomena.

## Introduction

How is science to explore and ultimately to understand the phenomena of everyday experience—things like inner speech, imagery, hearing, feeling, sensation, and so on? A dominant approach to understanding the neurophysiology of such phenomena is to deploy standardized tasks designed to elicit the phenomenon of interest and then to measure brain activations during task performance [1]. In a typical inner speech example [2], participants in an MRI scanner were instructed to listen to recorded sentences; brain activations were recorded when participants were cued to imagine covertly repeating those sentences to themselves. In a typical imagery example [3], participants were familiarized with line drawings of common objects; then in the MRI scanner brain activations were recorded when participants were cued to visualize one or another of those drawings.

A usually unstated assumption of such investigations is that on-demand tasks elicit brain activity and psychological/first-person/subjective phenomenology similar to spontaneous, naturally occurring phenomena, an assumption that allows findings about task-elicited experience to be generalized to naturally occurring experience. That assumption has not been adequately tested, and the ecological validity of task-elicited psychological states has not been established, particularly for phenomenologically complex experiences.

Some [4, 5] have questioned the elicited = spontaneous assumption. Regarding inner speech, there are at least four reasons to wonder whether elicited inner speech is the same phenomenon as naturally occurring inner speaking. First, the term “inner speech” typically (e.g., in [2]) refers to at least two phenomena, inner speaking and inner hearing, that are as phenomenologically and psychologically disparate as are speaking into a tape recorder and hearing your voice played back [6]. It seems plausible that such disparate phenomena could have distinctly different neural correlates [7]: whereas inner speech production is often associated with activation of left inferior frontal gyrus (IFG) [2, 8], “hearing” experiences may be expected to engage temporal lobe regions associated with auditory imagery, such as primary auditory cortex (Heschl’s gyrus) and superior and middle temporal gyri [9]. Second, there are large individual differences (ranging from near zero to near 100%) in the frequency of spontaneous inner speaking [6]; is inner speaking elicited from someone who essentially never spontaneously innerly speaks neurophysiologically and/or phenomenologically different from inner speaking elicited from someone who essentially always innerly speaks? Third, Jones and Fernyhough [4] argued that elicited inner speech such as required in [2] (‘repeat this [fully formed] sentence’) is *not* phenomenologically similar to natural inner speech, which is often abbreviated (omitting important words such as entire noun phrases), dialogic (conversational), and productive (not rotely repetitive). Fourth, spontaneous inner speech arises from intimately personal concerns, not external imposition.

Regarding visual imagery, there are large individual differences in the frequency of spontaneous imagery, differences at least as large as in inner speaking [5,10], and it seems plausible that the elicited imagery of people who nearly always spontaneously create imagery is neurally and phenomenologically different from the elicited imagery of people who never spontaneously create imagery. Furthermore, some elderly individuals’ spontaneous visual imagery is in black and white; however, when imagery is *elicited* from these same individuals, they produce color imagery (see ch. 9 in [5]). Thus the visual imagery that an individual *can* produce on demand may be substantially different from the visual imagery that that individual *does* produce spontaneously. Similar arguments could be made for potential differences between spontaneous vs. elicited hearing, feeling, sensation, and other experiential phenomena.

Empirical investigation of the comparability of the neurophysiology underlying elicited and spontaneous phenomena is difficult, however, both because investigating psychological phenomena is notoriously problematic [11] and because state-of-the-art neurophysiological measurement is cumbersome. Some [12–14] have developed questionnaires to inquire about spontaneous experience during the MRI resting state, but these questionnaires are retrospective; at best they characterize experience in general, not specific moments; and they do not attempt to overcome the difficulties of introspection that have been discussed [5, 11].

Some have used random probes, attempting to overcome questionnaires’ retrospectiveness and lack of focus on specific moments. For example, some researchers [15] have randomly probed participants during a boringly repetitive task and asked them to indicate whether they were attending to the task. Others [16, 17] have used the sound of the intermittent onset of the scanner itself as a cue to signal the reporting of ongoing experience, particularly auditory hallucinations, and used a subtractive method to compare brain activation during hallucinations to non-hallucination activation. However, there is reason to believe that participants’ experience-sampling responses may reflect their presuppositions about experience more than their actual experience that was ongoing at the sampling signal [18]. For example, Hurlburt, Heavey, and Kelsey [6] held that many people believe themselves to engage frequently in inner speech (a widely held belief about inner experience) and therefore in experience sampling studies frequently report that they are innerly speaking at the moment of the beep. However, more careful examination of their own randomly sampled moments reveals that inner speech is rare or nonexistent for them. Furthermore, some studies that seem aimed at spontaneous phenomena often do not actually investigate naturally spontaneous phenomena, instead investigating departures from a boringly repetitive task (e.g., [15]).

The present study uses Descriptive Experience Sampling (DES) [5, 19–23] to characterize the phenomenology of spontaneously occurring experience. DES, like other experience-sampling methods, uses a beep to probe experience, but DES is distinctive in that it endeavors to provide high-fidelity descriptions of whatever experience happens to be ongoing at the moment of the beep without having specified in advance what characteristics to explore. That is, DES seeks to describe whatever experience naturally, spontaneously happens to be ongoing.

To our knowledge, there have been no prior attempts to assess the brain activity of spontaneous phenomena that are naturally ongoing at specific moments in participants who have been trained to apprehend and describe phenomena in high fidelity. The present study takes an exploratory step in that direction, combining the neurophysiological sophistication of fMRI with the phenomenological sophistication of DES to compare task-elicited vs. spontaneously ongoing-at-the-moment experience. Combining those two procedures is not straightforward because DES studies are typically of extended duration (perhaps 12 to 24 hours of sampling) and take place in the natural environment, whereas fMRI studies are typically short (perhaps an hour) and take place in highly controlled environments.

To combine the two techniques, we first presented an orthodox fMRI task designed to elicit a variety of specific experiences (saying, seeing, hearing, feeling, or sensing something in imagination). Then we trained participants in DES in their natural environments, teaching them to set aside (“bracket”) [5, 24] their presuppositions about the nature of their own or others’ experience and to describe the features of whatever experiences happened to be “caught in flight” by the beep. Then, each participated in nine fMRI sessions which had no predefined task; each responded to 36 DES beeps (4 per session); each beeped experience was characterized using a DES expositional interview that was blind to the ongoing brain activation.

There were no a priori expectations about the characteristics of the experiences to be described at each beeped sample; for example, the study did not set out to target inner speech, or imagery, or hearing, or any other particular phenomenon. It turned out however that, according to DES, inner speaking was moderately common across our participants’ beeped experiences, allowing us to compare brain activations during spontaneous-inner-speaking moments to brain activations elicited by the orthodox inner-speaking task. To examine the potential difference between spontaneous and elicited inner speech, we focused on two regions of interest: the left IFG, based on its putative role in inner speech production [2, 25, 26, 27], and Heschl’s gyrus, which is part of the primary auditory cortex. Heschl’s gyrus was chosen because, in contrast to IFG, it is not usually considered part of any inner speech network but has been associated with auditory imagery and auditory processing more generally [9, 16]. Heschl’s gyrus is also clearly defined anatomically in comparison to other parts of posterior temporal cortex that have been associated with phonological representation in speech and imagery processing (such as the middle and superior temporal gyri [28, 29]). If elicited and spontaneous inner speech are similar, then strong activation in IFG but not Heschl’s gyrus would be expected in both conditions.

## Materials and Methods

### Participants

Ethics permission came from the Ethics Commission of the German Society of Psychology (SK 012013_5) according to the principles expressed in the Declaration of Helsinki. MRI time was to be provided by the Max Planck Institute for Human Development, so the study took place in Berlin. Interviews were to be led by RTH, the (English speaking) creator of DES, so we sought native English speakers. We recruited them by telephone from the databank of potential volunteers maintained by the Max Planck Institute. Informed consent was obtained both in writing and (repeatedly) orally. Given the substantial time and scanner commitment of this exploratory study, we determined a priori that we could apply the procedure to no more than seven individuals in the time available, so we recruited seven healthy volunteers. Two withdrew after the first sampling day, one for scheduling difficulties and one for unspecified reasons. The remaining five completed all phases of the study. All participants had normal or corrected-to-normal vision. No participant had a history of neurological, major medical, or psychiatric disorder. The participants (3 women and 2 men) had a mean age of 22.4 (ranging from 18 to 30). Four were right-handed; one male was left-handed (with laterality index -87.5 [30]).

## Procedure

### Design and overview

**Fig 1:**
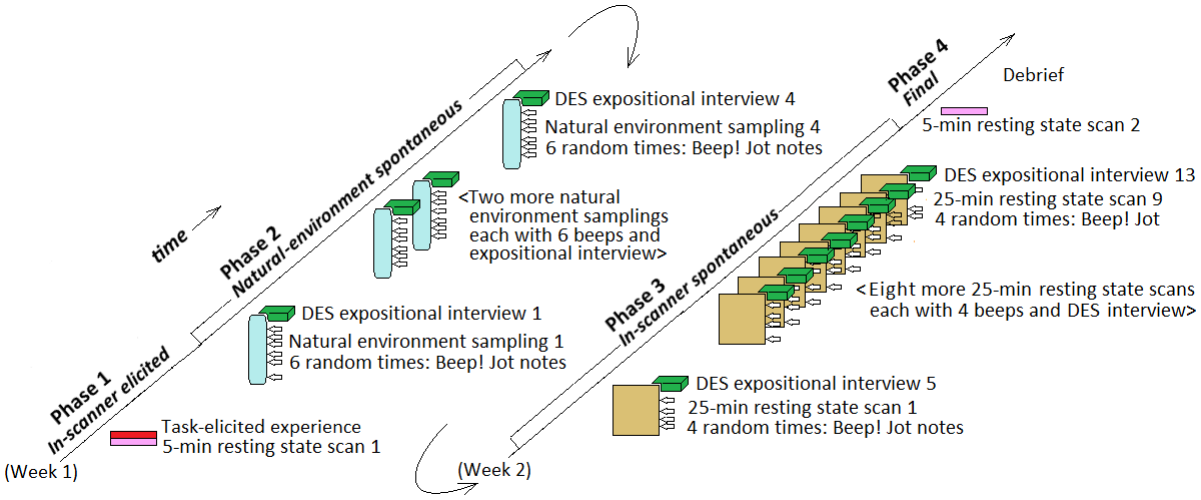
Schematic of the experimental design.

Each of the five participants was scheduled individually for 19 sessions, generally across a two-week period, which were divided into four phases as illustrated in Fig 1. In Phase 1 (*in-scanner elicited* phase, the first day of participation), we fully explained the study, administered a short battery of questionnaires not relevant to the present report, and familiarized the participant with the MRI scanner and procedures. The participant entered the scanner, where we conducted a 10 min structural scan and a 5 min resting state scan according to standard MRI research procedures (instruction: “please close your eyes and relax, without falling asleep”). Then we presented the elicitation task (adapted to include inner speech items from a recent fMRI imagery paradigm used by Belardinelli et al. [31]), where we visually presented short written prompts to imagine seeing (e.g., “to see a pencil”), saying (e.g., “to say ‘elephant’), hearing (e.g., “to hear a tinkling”), feeling (e.g., “the feeling of anxiety”), or sensing something (e.g., “the sensation of shiver”). Each category of prompt (seeing, saying, etc.) included 8 potential stimuli; the complete list of stimuli is in S1 Table. As described in Belardinelli et al. [31], the stimuli were presented in mini-blocks consisting of four prompts of one of the five categories, each shown for 7 s with 1 s inter-stimulus interval (= 32 s in total). Thus one mini-block consisted of four seeing prompts; another mini-block consisted of four saying prompts; and so on. Participants were instructed to vividly imagine what was requested by the prompt for the duration of the presentation of the prompt (7 s). After four prompts, a fixation cross was shown for 19 s before the next mini-block of prompts was presented. Each participant was presented with two mini-blocks for each of the five categories.

In Phase 2 (*natural-environment spontaneous* phase, typically beginning the first day and occupying the remainder of the first week), we instructed the participant in the use of the DES beeper and the sampling task [5, 18]: participants wore the beepers in their natural environments for approximately three hours, during which they would hear (through an earphone) six randomly occurring beeps. Immediately after each beep, the participant jotted down notes about whatever inner experience happened to be ongoing (was “in flight”) at the moment of the onset of the beep. Later that day or the next day, the participant returned for a DES expositional interview about those six beeped experiences; this interview was conducted by RTH and at least one other member of the research team including SK, BA-D, and/or CF as well as others. The expositional interview was “iterative” [5, 32], designed to increase skill in apprehending and describing inner experience across sessions. The participant then completed three more natural-environment sampling periods, each involving six random beeps, and each followed by an expositional interview. If communication about phenomena did not seem adequate after four sampling/interview days, additional sampling and interview sessions could be conducted; we invoked this option with one participant for one additional sampling period and expositional interview.

In Phase 3 (*in-scanner spontaneous* phase, typically occurring during the second week), the participant entered the scanner for a 25-min session with resting-state instructions (“keep your eyes open, stay relaxed and calm”). Otherwise, the participant’s experience was free to roam. At four quasi-random times, the participant received a DES beep through an MR compatible headphone (Visuastim), and then immediately jotted notes about the ongoing-at-the-moment-of-the-beep-onset experience on a clipboard positioned on the participant’s lap (viewable through a mirror). Immediately after exiting the scanner, we (RTH and at least one other interviewer, including SK, BA-D, and CF) conducted a DES expositional interview with the participant about the experiences that had been ongoing at the four randomly beeped moments. Thus the in-scanner sampling procedure was designed to be similar to the natural-environment sampling procedure. This sequence (25 min scan / four random beeps / expositional interview) was repeated a total of nine times (typically twice a day during the second week), resulting for each participant in 4 × 9 = 36 quasi-random samples of experience occurring in 25 × 9 = 225 minutes of fMRI scanning.

In Phase 4 (*final* phase), we administered a short battery of questionnaires not relevant to the present report. The participant was then candidly debriefed.

### Phenomenon selection

When an investigation aims at naturally occurring phenomena, the investigators cannot decide in advance what phenomena to study but must let the data dictate what will be (actually, already has been) investigated. In the present study we were prepared to study any of the five phenomena supposedly elicited by our modification of the Belardinelli et al. [31] tasks: seeing, saying, hearing, feeling, or sensing something. Sensory awareness was the most frequent in-scanner spontaneous phenomenon, occurring in 59 percent of the 180 samples; however, its varying modality (visual, bodily, auditory, etc.) made it an unlikely candidate for correlation with brain regions. Inner seeing was second most frequent in the scanner, occurring in 35 percent of the 180 in-scanner samples; however, inner seeing had zero frequency in two of the five participants’ natural environment DES sampling, and the correlation between inner seeing frequency in the natural environment and in the scanner for our participants was essentially zero (-.08); thus it seemed likely to be an artifact of the scanner situation. Inner speaking was next most frequent, occurring in 29 percent of the in-scanner samples; it occurred in all participants’ natural environment DES sampling and on at least 5 occasions for each participant in the scanner, and there was a high (.73) correlation across participants between the frequency of natural environment and in-scanner inner speaking, so inner speaking seemed a good candidate for further consideration. Inner hearing was rare (it occurred with appreciable frequency for only one participant, and occurred zero times or twice for three participants). Feelings were also rare (occurring in the scanner on only one occasion for three of the participants and never for the other two). Thus, as a result of the data that occurred, we focus here on inner speech-related neural processing, because there were enough such events to allow meaningful investigations.

### Scanning procedure

Images were collected on a 3T Magnetom Trio MRI scanner system (Siemens Medical Systems, Erlangen, Germany) using a 32-channel radio frequency head coil. Structural images were obtained using a three-dimensional T1-weighted magnetization-prepared gradient-echo sequence (MPRAGE) based on the ADNI protocol (www.adni-info.org) (repetition time [TR] = 2500 ms; echo time [TE] = 4.77 ms; TI = 1100 ms, acquisition matrix = 256 × 256 × 176, flip angle = 7°; 1 × 1 × 1 mm voxel size). Functional images were collected using a T2*-weighted echo planar imaging (EPI) sequence sensitive to blood oxygen level dependent (BOLD) contrast (TR = 2000 ms, TE = 30 ms, image matrix = 64 × 64, FOV = 216 mm, flip angle = 80°, voxel size 3 × 3 × 3 mm^3^, 36 axial slices, interleaved data acquisition).

### fMRI data pre-processing and main analysis

The fMRI data were analyzed using SPM8 software (Wellcome Department of Cognitive Neurology, London, UK). The first four volumes of all EPI series were excluded from the analysis to allow the magnetization to approach a dynamic equilibrium. Data processing started with slice time correction and realignment of the EPI datasets. A mean image for all EPI volumes was created, to which individual volumes were spatially realigned by means of rigid body transformations. The structural image was co-registered with the mean image of the EPI series. Then the structural image was normalized to the Montreal Neurological Institute (MNI) template using the segmentation-normalization procedure in SPM8, and the normalization parameters were applied to the EPI images to ensure an anatomically informed normalization. A commonly applied filter of 8 mm FWHM (full-width at half maximum) was used. Low-frequency drifts in the time domain were removed by modeling the time series for each voxel by a set of discrete cosine functions to which a cut-off of 128 s was applied. The statistical analyses were performed using the general linear model (GLM).

The elicitation task was modeled (following Belardinelli et al. [31]) as consisting of blocks with duration of 32 s length. In the spontaneous phase, the beeps of the DES procedure (i.e., instances of spontaneous inner speech) were modeled as events with a stick function on the onset of the beep and a duration of 0 s. Taking into account the standard 3 to 5 s delay in the hemodynamic response, this allowed us to model events in the brain in the seconds immediately prior to the moment of the beep. We also tried modeling with stick functions 1 s and 2 s before the beep onset and durations of 1 to 2 s for the “window” surveyed at each beep; these results were very similar to those reported below. These vectors were convolved with a canonical hemodynamic response function (HRF) and its temporal derivatives. Then, for the DES procedure, regressors were built coding the categories that raters (RTH with at least one other) assigned to the individual’s 36 events.

### ROI analysis

To investigate inner speech, we selected two anatomical ROIs taken from the Automatic Anatomical Labeling (AAL) atlas [33]; left inferior frontal gyrus (IFG), because of its association with speech production in right handers, and Heschl’s gyrus, i.e. primary auditory cortex. Although some studies have focused on specific subsections of the IFG such as BA 44 (e.g., [2]), we included the whole IFG based on the fact that previous investigations of inner speech have often included multiple Brodmann Areas [8, 27, 34]. We used right IFG for the left handed participant, whose laterality index was -87.5 and who clearly showed contralateral activation during speech production in a whole-brain analysis. Furthermore, the results are similar if we exclude this participant. Separately for each participant, each anatomical ROI, and each condition, the beta weights from the model detailed above were extracted [35] and used for further analysis. We chose these ROIs because any difference between spontaneous and elicited inner speech might show up as a different patterning of activation across either or both speech production and auditory perception areas. The results presented below are largely independent of the exact definition of the auditory ROI, because similar results were found when using a ROI spanning the functionally defined primary auditory cortex.

### DES coding

Within 24 hours of each DES expositional interview, one of the interviewers wrote a description of each of that day’s samples. These descriptions were then circulated to the others for comment, with any disagreement resolved or left as an explicit disagreement, usually within 48 hours of the original interview. RTH and at least one additional person present at the interview independently judged whether any (or several) of the five phenomena designed to be elicited by the Belardinelli et al. [31] tasks (seeing, saying, hearing, feeling, or sensing something) was present at each sample; discrepancies were resolved by discussion. These ratings were thus constrained by the descriptions: the descriptions had been written and agreed to before the rating process began. However, the ratings were not intended to be ratings *of the written descriptions*; they were intended to be ratings *of the experiences that had occurred at the moment of the beep*. On a few (approximately six) occasions, the rating procedure highlighted an ambiguity, omission, or misleading aspect of the written description, in which case a return to the interview videotape might result in a revision of the written description. All such adjustments of the written descriptions were performed blind to the brain activations and analyses thereof. Idiographic characteristics (those that emerged distinctively for particular participants) were also described, but are not discussed here. Ratings for each participant are found in S2–S6 Tables.

## Results

**Fig 2:**
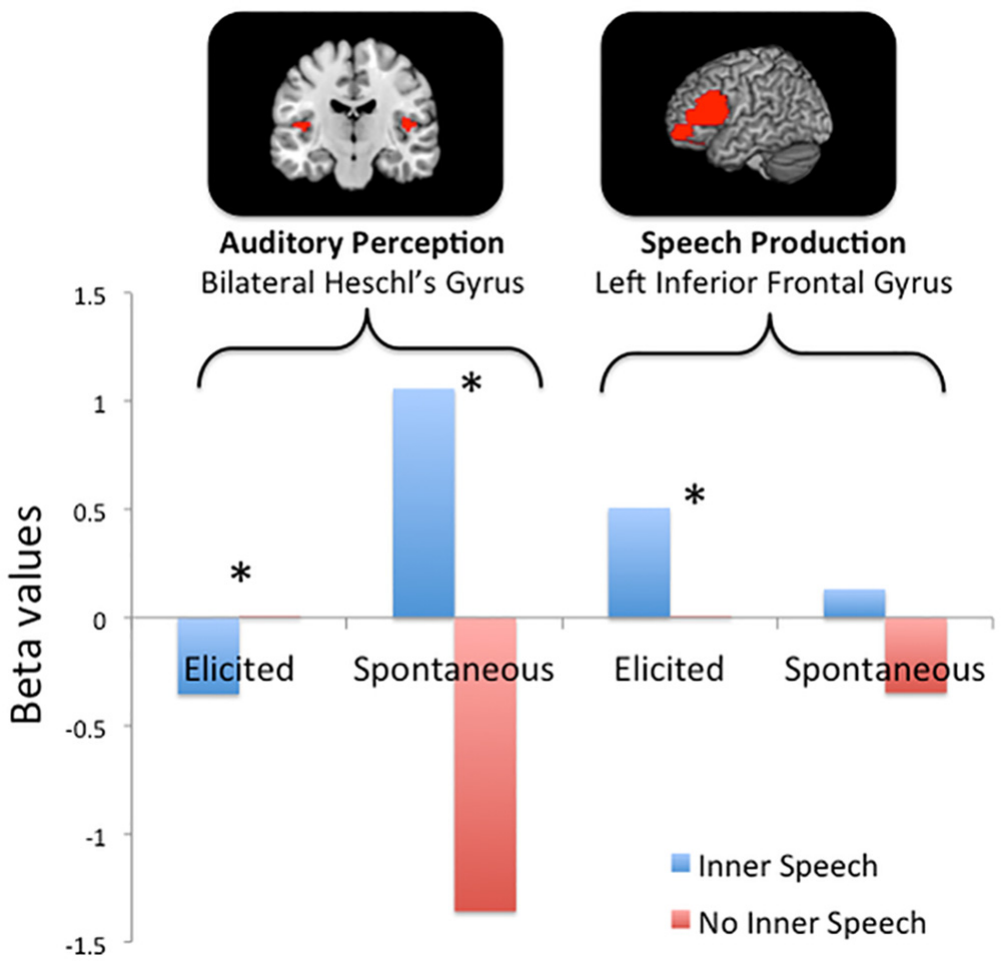
Beta-values extracted from brain regions associated with auditory perception (Heschl’s gyrus) and speech production (left inferior frontal gyrus). In the elicited condition, speech was prompted by means of visual cues. In the spontaneous condition, Descriptive Experience Sampling was used to capture naturally occurring moments of inner speech.

Phase 1 (*in-scanner elicited* phase) involved orthodox elicitation tasks including cueing participants to imagine silently speaking words such as *lamp*. The results are shown in the *elicited* bars in Fig 2. Elicited inner speaking showed significant deactivation in bilateral Heschl’s gyrus (auditory perception), *t*(4) = -2.96, *p* = .04, and significant activation in left IFG (speech production), *t*(4) = 3.00, *p* = .04, when elicited inner speech was compared with the intercalated fixation periods. We therefore conclude that our inner-speech elicitation task produced activation patterns similar to those found in other laboratories.

In Phase 3 (*in-scanner spontaneous* phase), participants provided 180 beeped samples, of which 52 (29%), according to the DES expositional interview, included inner speaking ongoing at the moment of the beep (across participants, the mean number of inner speaking samples was 10.4, SD = 5.94, range = 5–19). We considered the brain activity in the two ROIs for these 52 samples compared to implicit baseline and discovered a significant difference in Heschl’s gyrus (*t*(4) = 4.27, *p* = 0.013) and none in left IFG (*t*(4) = 0.188, *p* = 0.86)

The putative fidelity of the DES apprehensions of phenomena allowed us to ascertain that those 52 inner speaking samples included occasions where inner speaking was just one of several simultaneous ongoing characteristics of experience (e.g., inner speaking while simultaneously seeing an image); in fact, in many samples, imagery or some other phenomenon or phenomena were experientially dominant while inner speaking was experientially of only secondary or tertiary importance. Furthermore, there were other samples where the occurrence of inner speaking was somewhat questionable (e.g., the participant reported something like “*I think* I was speaking that to myself, *but I’m not sure*”). It is therefore possible that the observed decreased activation in Heschl’s gyrus was due not to inner speaking per se but to some other phenomena that accompanied or perhaps dominated inner speaking. We therefore reconsidered the original 180 sampled experiences, seeking those samples where inner speaking was clearly dominant and those other samples where inner speaking was clearly absent. In 20 (11%) of them, three raters (RTH, CF, and BA-D working independently) were unanimously confident that inner speaking was (according to DES) the predominant feature of the experience (across participants, mean = 4.0, SD = 2.92, range = 0–7). The same three raters also independently and unanimously identified 85 (47%) samples where inner speaking was confidently *not present* at the moment of the beep. As a result of this classification, it was possible to contrast the brain activity ongoing at the 20 predominantly inner speaking moments with the 85 samples of brain activity where inner speaking was confidently *not* present. The results were similar to and in the same directions as those found for all the spontaneous inner speaking samples: the contrast for Heschl’s gyrus was significant, *t*(4) = 6.04, *p* = .004; the IFG contrast was not significant, *t*(4) = 0.74, *p* = .50. These results are shown in the *spontaneous* bars in Fig 2 for the Heschl’s gyrus and left IFG regions of interest.

The question of primary interest is whether elicited inner speech and spontaneous inner speech involves different neural activations. The ROI × task (spontaneous vs. elicited) interaction was significant, *F*(1, 4) = 9.26, *p* = .04, as was the ROI × task × condition (inner speech vs. no inner speech) *F*(1, 4) = 37.21, *p* = .004, suggesting differential activations of the two ROIs by elicited and spontaneous processes. Elicited inner speech was associated with a significant *decrease* in activity in Heschl’s gyrus and a significant increase in left IFG. By contrast, spontaneously-occurring inner speech was associated with a significant *increase* in activity in Heschl’s gyrus but no significant change in left IFG. The apparently dissociated pattern (decreased activation when elicited but increased activation when spontaneous) in Heschl’s gyrus was also seen in each of the participants individually.

## Discussion

In an intensive investigation of five participants, we prepared ourselves to compare neural activations accompanying task-elicited phenomena (inner seeing, inner speech, inner hearing, feeling, and/or sensing) to putatively similar naturally occurring phenomena. Because of the characteristics of the naturally occurring phenomena, we focused on the comparison of a standard elicited-inner-speech task to activations accompanying spontaneous inner speaking. Consistent with prior studies, task-elicited inner speech was associated with increased activation in left IFG; alongside this, deactivation in primary auditory cortex was also evident (Heschl’s gyrus). However, for spontaneous inner speaking a contrasting pattern occurred: inner speaking was associated with *increased* activation in Heschl’s gyrus and no evidence of change in left IFG, suggesting a difference between task-elicited and spontaneous inner speech.

### Neurophysiological considerations

We observed that elicited inner speaking caused a decrease in activation of Heschl’s gyrus, in accord with prior elicited-inner-speech research and with some understandings of external speech, which hold that when we talk, there is a reduction in sensitivity to our own speech sounds [36]. External speaking is often associated with the dampening of neural responses to expected sounds in auditory cortex [37, 38], and similar effects are evident during silent articulation [39], suggesting that articulatory processes are sufficient to induce auditory suppression even in the absence of external speech. Similarly, in the silent-speech case, it may be that elicited inner speech involves a sufficient level of articulation—albeit silent—to inhibit responses in Heschl’s gyrus.

By contrast, we found that spontaneous inner speaking was associated with an increase in Heschl’s gyrus activation. That is the more surprising because inner speaking is phenomenologically *speaking*, not *hearing* [6]. One interpretation of these results is that perhaps Heschl’s gyrus is more involved in representing speech than is usually credited. Though more commonly linked with non-verbal auditory imagery [40], Heschl’s gyrus activation is also evident during silent lip-reading [41] and in some cases of auditory verbal hallucinations [42]. Surrounding areas of middle and superior temporal cortex often activate during voice imagery tasks [17, 43], and similar processes could have driven the activation patterns observed here.

Alternatively, the activation of left IFG by task-elicited inner speech may reflect more the elicitation task demands than the speech itself. Though classically linked to speech production, left IFG is also thought to be integral to planning and execution of hierarchical sequences and complex actions more generally [44, 45]. Task-elicited inner speech, unlike spontaneous inner speaking, also involves the reading of the command, its decoding, and the acquiescence thereto as well as the covert speaking of the commanded words. That we did not find evidence of IFG involvement during the spontaneous inner speech task may be the result of insufficient power to detect IFG involvement or the result of a cognitive difference between elicited and spontaneous inner speaking.

The small-*n* of this study makes it possible that our 5 participants were an unusual group, not representative of the wider population, and so our neurophysiological findings may have limited generalizability. However, this is made unlikely by two observations. First, our results did replicate the usual findings for task-elicited inner speaking, so our participants are likely to be not grossly dissimilar from the wider population. Second, the Heschl’s gyrus reversal (decrease when elicited, increase when spontaneous) was found for each of the five participants individually. Our participants were a heterogeneous group: male and female, right and left-handed, ranging in age from 18 to 30, all unacquainted with each other. They were not, seemingly, a very specialized group. On the other hand, they were all Caucasian native English speakers, so we can make no cross cultural claims.

### Methodological considerations

The importance of this study arises as much from the exploration of method as from the exploration of neurophysiology. We turn to that discussion now.

#### Sample size and power

For reasons discussed above, the present study used a small number (5) of participants. There are two separable disadvantages of small-*n* studies: generalizability and power. Regarding generalizability, the small-*n* study makes it possible (although unlikely as we have seen) that our 5 participants were not representative of the wider population. However, even granting the possibility that our group of participants did have some unusual characteristics, the results still suggest that the elicited/spontaneous inner speaking distinction is important at least for some people, raising important questions for further research: What kinds of people? Under what circumstances? Using what methods?

Regarding power, we note that power depends on effect size and sample size in the numerator and experimental error (unknown off-task behaviors and extraneous characteristics of individual participants) in the denominator. Typical studies use a large numerator (principally a large sample size) to obtain sufficient power to overcome large denominator experimental error. By contrast, the spontaneous phase of the present study attempted to create a small denominator (by investigating homogeneous phenomena) to overcome the small numerator (the result of the small sample size). We did this in two steps. In the first step, we provided participants far more practice in describing their experience than is usual in psychological studies. This skill-building practice involved four days of DES sampling in their natural environments, each with an intensive DES expositional interview. Those interviews are iterative [5, 32], explicitly designed to improve, over the course of the successive sampling days, both the participant’s skill in apprehending and describing inner experience and the investigator’s skill in understanding what the particular participant intends by perhaps idiosyncratic locutions. Among this clarification of many characteristics of inner experience, that practice is intended to clarify (of particular relevance here) what is and is not inner speaking. Without such skill-building practice, what an experimental participant classifies as “inner speaking” can vary greatly from participant to participant [6, 18], and thus typically contributes to large experimental error. The DES iterative process was designed to reduce that error by ensuring (as best as can be done with respect to private experience) that when the DES investigator noted that inner speaking was occurring at the moment of some beep, a consistent definition of “inner speaking” was being used across participants. This intent to reduce the denominator by investigating relatively homogeneous phenomena was successful in Heschl’s gyrus: we found a significant result (*t*(4) = 4.27, *p* = 0.013) even with the small sample of 5 participants and 52 samples.

The second step in the attempt to reduce the denominator involved making the denominator even more homogeneous. The putative high fidelity of the DES interviews allowed us to notice that even within those samples identified as including inner speaking, there remained substantial variability. For example, at a sample in her fourth scanner session, “Susan” was innerly seeing an image of her hand breaking away from her dad’s hand when she was about 13, and was simultaneously feeling how her dad must have felt when she stopped holding his hand; and was simultaneously innerly speaking in her own voice, “it’s natural, she’s growing up.” In an investigation of the brain correlates of the phenomenon of inner speaking, the brain correlates of Susan’s vivid visual imagery and of her empathic feeling are experimental error. We therefore sought to reduce these kinds of error by including only instances where inner speaking was unambiguous and predominant (excluding instances where extraneous phenomena were dominant or where identification of phenomena was problematic). This approach also led to a significant result in Heschl’s gyrus (*t*(4) = 6.04, *p* = .004) even though the number of brain samples considered was much smaller (20 samples where inner speaking predominated compared with 52 samples where inner speaking was present but not necessarily strong).

This study’s two approaches to investigating relatively “pure” experiential phenomena are novel and thus demonstrate potential new avenues for investigative approaches. We do not claim that one approach is superior to the other; however, we do believe we have demonstrated that the two approaches deserve more research aimed at ascertaining under what circumstances the attempt to refine phenomena is justified and effective.

#### Task validity

The inner-speech elicitation task we used here involved simple repeat-one-word inner speech; however, it could be argued that one is unlikely to innerly say a single word in spontaneous inner speaking. Might our Heschl’s gyrus results be due to differences in content (one word vs. many) rather than to the distinction between elicited and spontaneous experience? More research using a variety of elicitation paradigms (e.g., rhyming judgments [46] or dialogues [47]) could demonstrate what difference the form and length of inner speech might make. However, we think that the design of the present study makes this objection unlikely. First, it is not implausible to suggest that spontaneously occurring inner speech might sometimes involve single words, due to processes such as condensation [48]. Second, this study did not compare directly elicited and spontaneous inner speech under the same conditions. It compared elicited inner speech to baseline in one session (left bars in the panels of Fig 2), and separately compared spontaneous-inner-speech to spontaneous-*not*-inner-speech in other sessions (right bars in the panels of Fig 2). The involvement of left IFG (and relative lack of activation in Heschl’s gyrus) in our speech-elicitation task is similar to the findings of other studies that use a variety of inner-speech-elicitation methods including longer sentences and rhymes (e.g. [21]). The logic of the present study uses that result to show that the brain activations of our participants are what one might expect, suggesting that differences in utterance length are unlikely to account for the observed differences in activation for elicited and spontaneous inner speech. Thus the nature of the elicitation task is not necessarily the most important feature of this study.

We note that it would be desirable to make the comparison between elicited and spontaneous inner speaking more direct. For example, in a multiple-session MRI design, it would be theoretically possible to capture a spontaneous instance of inner speaking in session 1 and then, embedded in session 2, request the participant to innerly speak a sentence that has been crafted to resemble the session 1 instance. Such designs are desirable, and we encourage researchers to pursue them (while at the same time noting that they present substantial practical obstacles, not least of which being that such an elicitation task would directly disturb the spontaneous environment).

#### Studying spontaneous phenomena

This study demonstrates that it is possible to investigate spontaneous experience in the scanner without *a priori* specification of the phenomenon under consideration, thus avoiding a potential biasing of responses. Nearly all fMRI studies specify in advance the phenomenon to be investigated, either by requiring participants to perform a specified task or by instructing the participant to press a button when the specified phenomenon occurs (a possible exception is Shergill et al. [16, 17], but those reports do not provide enough information to be confident about how the interview was conducted). By task or by button-press instructions, investigators thus are instructing participants to focus on some particular predefined aspect of behavior or experience. Such a focus has at least the potential to bias or distort the experience or its underlying physiology; the conditions under which such bias/distortion is important have not been adequately investigated. This study demonstrates that such investigation is possible.

Investigating spontaneous experience requires an intensive method (here, 11 scanner sessions, 13 sampling periods and associated expositional interviews per participant). Such studies will perforce have a smaller-than-typical number of participants (potentially balanced, as we have seen, by a higher-than-typical fidelity of experiential report).

Investigating spontaneous experience makes individual differences an unusually salient feature of the research process. Most neurophysiological research ignores individual differences. For example, when Simons et al. [2] investigated inner speech, they recruited participants without regard for their inner experience characteristics. They instructed them to listen and covertly repeat sentences, apparently under the (unstated) assumption that inner speech processes are universals or that individual differences are inconsequential features that would average away across many participants. By contrast, we investigated inner speaking because it turned out that our careful observations revealed that our participants frequently spontaneously innerly speak. Had spontaneous inner speaking not been frequent, we would not have studied inner speaking. Thus our results might be stated: elicited and spontaneous inner speaking differ *among those who spontaneously innerly speak*. However, many people rarely or never spontaneously innerly speak [6]. Our results shed no light on whether the neurophysiological responses of individuals who rarely or never innerly speak might differ from those who innerly speak frequently; there is nothing that we know of in the literature that addresses this point.

#### Fidelity

This study highlights the importance of bracketing presuppositions. As we observed above [6], many people (including, apparently, our participants) erroneously but deeply and without examination believe that inner speaking is ubiquitous. Prior to any participation, we asked our participants to estimate the proportion of time they themselves typically engaged in inner speaking; their percentages averaged 72 percent. However, their natural environment inner-speaking sampling frequency averaged just 18 percent, in line with Hurlburt’s [5, 18] suggestion that people’s retrospective or general accounts of their experience should not be taken at face value. That conclusion applies when the retrospection is performed immediately after exiting the scanner unless the retrospection is constrained (as here) by notes taken immediately after the experience occurred [49].

This study highlights the potential importance of the fidelity of the sampling method. Most experience-sampling studies do not adequately train their participants to make the discriminations that their research requires—something like an iterative process is required for such training [18]. The present study was very careful in that regard, allowing us to select samples where inner speaking clearly predominated. We suggest that there are two broad strategies for dealing with the fidelity of most experiential reports: (a) to accept that experiential reports are of low fidelity and therefore employ a large number of participants on the assumption that the low-fidelity-causing distortions are largely stochastic and will thus average away across participants (an assumption that is rarely evaluated, and which has been criticized [18]); or (b) to provide high fidelity descriptions by using a small number of participants who are adequately trained and a procedure that is adequately implemented. There are many studies of kind (a); the present study is an example of the (rare) kind (b). Both approaches have their advantages and disadvantages; further research is required.

#### Differences between elicited and spontaneous experiences

It should perhaps not be assumed that people actually innerly speak when instructed so to do, even though in debriefing they typically sincerely aver that they had complied with the instructions. That is, “task elicited inner speech” in some, perhaps many, instances does not actually involve inner speaking. Many DES participants early in sampling believe themselves to have been innerly speaking frequently when beeped, whereas subsequent interviews conclude that those experiences had more likely been examples of sensory awareness, unsymbolized thinking, images, or other non-speech phenomena [6]. This over-reporting of inner speech follows from presuppositions, including the erroneous belief that inner speaking is ubiquitous [6].

We have concluded that it is likely that elicited inner speech differs from spontaneous inner speaking for at least some people and some elicitation tasks. We do not speculate about whether the elicited/spontaneous distinction might apply to other inner phenomena such as visual imagery (are elicited images the same as spontaneously occurring visual images?) or to cognitive processes that are not themselves typically the target of experience. There may be something unique about the experience of speaking that makes the distinction between elicited and spontaneous inner speech more salient than for other phenomena or processes. Nevertheless, the present study suggests that exploring the elicited/spontaneous distinction for imagery (and for other phenomena) might be fruitful avenues for further research.

Meeting the challenges involved in studying spontaneous phenomena would seem well justified, given that much psychological theorizing overlooks the distinction between task-elicited and spontaneously occurring phenomena. For example, auditory verbal hallucinations (AVH) have been hypothesized to result from a misattribution of inner speech to an external source [50, 51], a theory that is based largely on the fact that left IFG is activated both in neuroimaging studies of AVH (see meta-analyses in [52, 53]) and in neuroimaging studies of elicited inner speech [8]. Thus the overlap of activations in *elicited* inner speech and AVH have been used to support neurocognitive models of AVH as involving aberrant monitoring of *spontaneous* inner speech ([54]). Whether task-elicited phenomena are neurally and phenomenologically similar to spontaneously occurring phenomena is therefore an open question with important implications.

In sum, we conclude that elicited inner speaking should not be assumed to be the same as spontaneous inner speaking. This is potentially of fundamental importance if there is to be any prospect of studying everyday inner experience using standard neuroimaging methods: studies that attempt to elicit experience should recognize that their observed neural correlates may not be the same as those obtained when investigating natural phenomena. In the specific case of inner speech, much of science’s view of inner speech is based on task-elicited paradigms; understanding of inner speech’s underlying functional anatomy might be substantially altered if those studies were supplemented by explorations of spontaneous phenomena.

## Supporting Information

S1 Table Elicitation task prompts. (DOCX)

S2 Table Participant 1 data ratings. (XLSX)

S3 Table Participant 2 data ratings. (XLSX)

S4 Table Participant 3 data ratings. (XLSX)

S5 Table Participant 4 data ratings. (XLSX)

S6 Table Participant 5 data ratings. (XLSX)
