# Supplementary material for: Exploring the Ecological Validity of Thinking on Demand: Neural Correlates of Elicited vs. Spontaneously Occurring Inner Speech
Source: PLoS One. 2016 Feb 4;11(2):e0147932. doi: 10.1371/journal.pone.0147932 (PMC4741522; doi:10.1371/journal.pone.0147932)
Supplement: S1 Table — (DOCX) [file pone.0147932.s001.docx]

**S1 Table: Elicitation task prompts**

**Seeing:**

To see a bulb

To see a coin

To see a book

To see a candle

To see a pencil

To see a tree

To see a house

To see a cloud

**Saying:**

To say “car“

To say “elephant“

To say “pencil“

To say “book“

To say “chair“

To say “lamp“

To say “bicycle“

To say “phone“

**Hearing:**

To hear a whistle

To hear an uproar

To hear a chime

To hear s.o. talking

To hear a rustle

To hear a conversation

To hear a tinkling

To hear s.o. yelling

**Feeling:**

The feeling of anxiety

The feeling of happiness

The feeling of joy

The feeling of fear

The feeling of nervousness

The feeling of embarrassment

The feeling of sadness

The feeling of anger

**Sensing:**

The sensation of pain

The sensation of shiver

The sensation of smelling gasoline

The sensation of itch

The sensation of stiffness

The sensation of fatigue

The sensation of creeps

The sensation of exertion
